# Vibration and Noise Transmitted by Agricultural Backpack Powered Machines Critically Examined Using the Current Standards

**DOI:** 10.3390/ijerph16122210

**Published:** 2019-06-21

**Authors:** Angela Calvo, Christian Preti, Maria Caria, Roberto Deboli

**Affiliations:** 1 DISAFA (Department of Agricultural, Forest and Food Sciences and Technologies), Largo P. Braccini 2, 10095 Turin, Italy; 2 IMAMOTER Institute for Agricultural and Earth-moving Machines of C.N.R (Italian National Research Council), Strada delle Cacce 73, 10135 Turin, Italy; c.preti@ima.to.cnr.it (C.P.); r.deboli@ima.to.cnr.it (R.D.); 3 Dipartimento di Ingegneria del Territorio, Sezione di Meccanizzazione e Impiantistica, Viale Italia 39, 07100 Sassari, Italy; mariac@uniss.it

**Keywords:** vibration, noise, blower, mist blower

## Abstract

European Directives 2002/44/EC and 2003/10/EC establish the exposure limit values for preventing operators’ risks to vibration and noise transmitted by machines. Few studies studied noise and vibration of agricultural backpack powered machines (as mist blowers and blowers), but nobody critically studied them. This work analyzed the field back vibration, hand-arm vibration (HAV), and noise transmitted to ten operators by eight blowers and mist blowers. Unweighted and weighted vibration were analyzed, using the standards ISO 2631-1 (back), and ISO 5349-1 and ISO/TR 18570 (hand-arm system). The noise was evaluated by recording the acoustic pressure level at the operators’ ears using the ISO 9612. With the ISO 2631-1, the vibration to the operators’ back was low (0.38 ms^−2^), but the unweighted vibration measured along *y* and *z*-axes (not used by the ISO 2631-1) were high (>11 ms^−2^). HAV were also low when using the ISO 5349-1 (the highest value was 2.51 ms^−2^ in mist blowers), but high with the ISO/TR 18570 for the onset of vibration white finger (1446 ms^−1.5^ in blowers). Noise levels were always high: more than 100 dB(A), excluding the blower with the exhaust inside the blower hose. This last machine had noise levels lower than 86 dB(A), but its specific feature could increase environmental pollution.

## 1. Introduction

European Directives 2002/44/EC and 2003/10/EC [1,2] establish the exposure limit values and the exposure action values for preventing operators’ risks to vibration and noise transmitted by machines and powered tools. These Directives use the current standards for vibration and noise measurements [3,4,5], and the employers use them for the risk evaluation.

In agriculture, there are many hand-held and backpack powered machines that may transmit a high level of noise and vibration to the operators. Loggers, gardeners, and many farmers, in fact, frequently use chainsaws, brush cutters, hedge cutters, blowers, and mist blowers. The intrinsic characteristics of these machines for professional use (many of them are petrol engine and their mass usually never exceed 12 kg) may cause physical risks (noise and vibration) to the operators. Many studies concerned the hand-arm vibration (HAV) and the noise risks caused by chainsaws [6,7,8,9,10,11] and brush cutters [12,13,14,15,16,17,18]. Some authors focused their studies on noise and HAV caused by hedge cutters and blowers [7,19,20], while others were interested in the physical risks produced by mist blowers [21,22,23,24].

Blowers and mist blowers are backpack powered machines, carried on the operator’s back by a harness. The blower is a “ducted fan” that blows air at high speed from a nozzle, and it is used to pile up leaves, grass clippings, and litter. The mist blower is used for the application of insecticides or fungicides as a mist. The equipped fan produces a great volume of air, and a pipe conveys it to a nozzle located at the bottom of the throwing pipe. Blowers are widely used by municipalities for cleaning streets (never less than 2 h per day, [14]), while mist blowers are used in farms with little crops and the daily use depends on the geographic context: for example, Denkyirah et al. [25] found that, in Ghana, they are used at least 6 h per day. Both the machines produce high levels of noise and transmit vibration to the operator’s body on both the back and the hand-arm system.

This work studied vibration (back and HAV) and noise transmitted to ten operators by eight different mist blowers and blowers. Back and HAV vibration were studied using both raw unweighted and weighted data (the last using the current standards): the use of unweighted acceleration was useful to appreciate some constraints in the current standard used for evaluating back vibration and to enforce the use of the new proposed standard for the HAV analysis. 

## 2. Materials and Methods

Back vibration was previously studied using unweighted acceleration, and, therefore, it was used as the *W_c_* weighting curve, as requested by the ISO 2631-1 for the health evaluation (the *W_c_* weighting curve only takes account of the *x*-axis) [3]. HAV produced by blowers and mist blowers were formerly studied without filtering the accelerometer output (at the operators’ handle): afterward, the *W_h_* and *W_p_* weighting curves (as indicated, respectively, by the ISO 5349-1 [4] and the ISO/TR 18570 [26]) were used. The noise was calculated by recording the equivalent continuous A-weighted sound pressure level (measured at the operators’ ears), according to the ISO 9612 [5].

Four blowers and four mist blowers were tested in the field. Ten experienced, right-handed male operators were involved (Table 1). All runs were performed in September 2018, in the fields of the DISAFA campus (Department of Agricultural, Forest, and Food Sciences and Technologies) located in Grugliasco (Torino, Italy; GPS: E 7.3446, N 45.0354). During the runs, the weather was sunny, the temperature was between 20 and 24 °C, and the speed of the wind was between 2.3 and 3.7 ms^−1^. All runs were performed at low (LES) and at high engine speed (HES): the LES phase occurred when operators were approaching the crop, while the HES phase concerned the operators’ work during spraying or blowing. Three series of runs were executed for each machine test, in both vibration and noise analysis.

### 2.1. The Examined Machines

**Figure 1:**
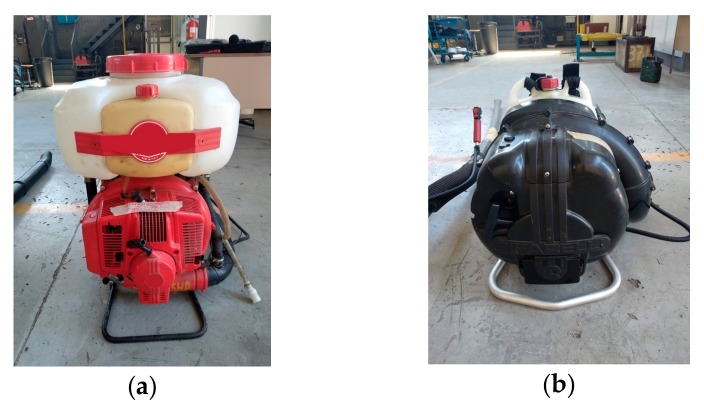
Two tested machines (a mist blower (**a**) and a blower (**b**)).

Four mist blowers and four blowers, single-cylinder, two-stroke, and air-cooled engines, were tested (Figure 1). The machines had padded backrests and belts easy to adjust. Anti-vibration materials were present between the backrest frame and the engine-fan system. The machines had an average use of 300–350 h, and the tanks of the mist blowers were filled with 3 liters of pesticide (Table 2).

### 2.2. Vibration

#### 2.2.1. Measurement Chain

**Figure 2:**
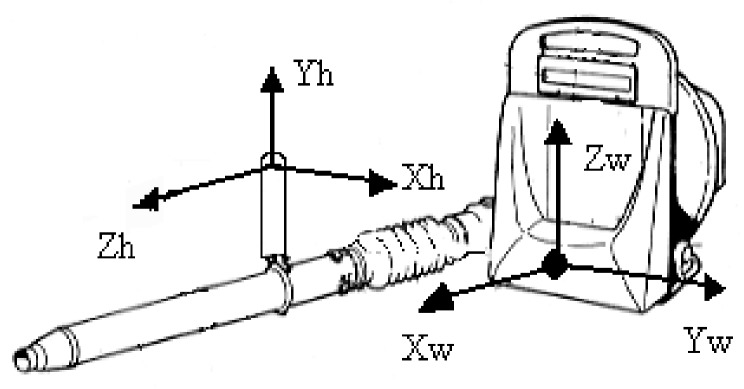
*x*, *y*, and *z*-axes of the three-axial accelerometers on the padded backrest (in figure—*Xw*, *Yw*, and *Zw*) and on the control handle (in figure—*Xh*, *Yh*, and *Zh*).

The real-time acquisition of the back vibration was performed by a tri-axial accelerometer ICP (Integrate Current Preamplifier), model 356B41 (PCB Piezotronics, Depew, NY, USA), sensitivity 100 mVg^−1^, and mass 10 g. Measurements were carried out along the three axes: *x* (fore-and-aft direction), *y* (shoulder-shoulder direction), and *z* (buttocks-head direction, Figure 2). 

**Figure 3:**
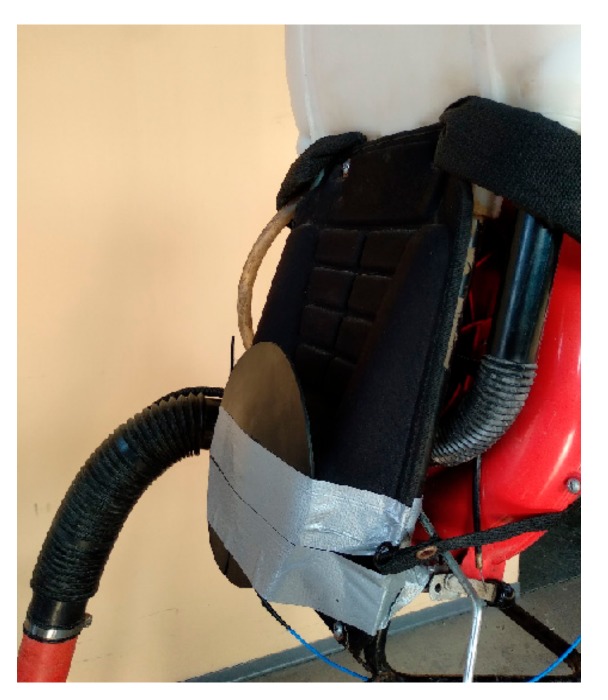
Position of the rubber pad containing the accelerometer, on the padded backrest.

The accelerometer was inserted in a rubber pad, fixed by adhesive tape on the bottom of the padded harness [27] (Figure 3). A tri-axial accelerometer (ICP, model PCB SEN020, 1 mVg^−1^ sensitivity, 10 g mass) was fixed at the control handle near the power switch (using a metallic screw clamp) for the vibration measurement on the hand-arm system [28]. The head of the third metacarpal was the origin of the system. The *x*-axis was perpendicular to the palm area (positive in the back direction), the *y*-axis, perpendicular to the *x*-axis, passed through the origin, and the *z*-axis was longitudinal to the third metacarpal (Figure 2).

The accelerometers were calibrated by a Brüel & Kjær calibrator, type 4294 (standard acceleration level 10 ms^−2^), and the measurement system was checked before each set of runs. Acceleration signals were sent to a National Instruments data acquisition card (NI 9234, sampling rate 51.2 kSs^−1^—kilo samples per second—for each channel). The acquired signals were processed using the LabVIEW software (V.12.01f5, National Instr. Corp., Austin, TX, USA) to obtain unweighted one-third octave band magnitudes. Signals were, afterward, frequency weighted with the weighting curves [3,4,26].

#### 2.2.2. Back Vibration: Unweighted and Weighted VTVs (Vibration Total Values)

The ISO 2631-1 standard uses only the *W_c_* weighting curve for the *x*-axis and the weighting factor 0.8 to evaluate the health at the back (*y* and *z*-axes are not considered in this standard for the health evaluation). The vibration total values (VTVs) *a_v_* (ms^−2^) were the root mean square (r.m.s.) of both the unweighted and weighted *W_c_* accelerations (1).

(1) $$a_{v}=\sqrt{k_{x}^{2}a_{wx}^{2}+k_{y}^{2}a_{wy}^{2}+k_{z}^{2}a_{wz}^{2}}\;({\mathrm{ms}}^{-2})$$

where *a_wx_*, *a_wy,_* and *a_wz_* were acceleration unweighted and *W_c_* weighted for the health analysis, and *k_x_, k_y_, k_z_* were multiplying factors (*k_x_, k_y_, k_z_* = 1 when unweighted; *k_x_* = 0.8 and *k_y_,k_z_* = 0 when *W_c_* weighted), according to the ISO 2631-1. The vibration daily exposure *A(8)* (ms^−2^) was calculated (2) and compared with the daily exposure action value (0.5 ms^−2^) and with the daily limit value (1.15 ms^−2^), according to [1].

(2) $$A(8)=a_{v}\sqrt{\frac{T}{8}}\;({\mathrm{ms}}^{-2})$$

where *T* (h) was the daily use of the vibrating machine, and 8 (h) was the daily working hours (according to [1]).

#### 2.2.3. HAV: Unweighted and Weighted VTVs

Accelerations *a_x_*, *a_y_*, and *a_z_* were simultaneously acquired along the three perpendicular axes for the hand-arm system [28]. Each measurement was two min long. The time signals from the accelerometers were processed to obtain the one-third octave band magnitudes. Unweighted and frequency weighted accelerations were acquired, using the *W_h_* weighting curve (as requested by [4] for bones, joints, and muscles) and the *W_p_* weighting curve (as set by [26] for the vascular system). The application of the *W_h_* curve produced the frequency-weighted accelerations *a_hwx_, a_hwy,_* and *a_hwz_*. The VTVs (*a_hv_*, ms^−2^) were calculated as their r.m.s. (3).

(3) $$a_{hv}=\sqrt{a_{hwx}^{2}+a_{hwy}^{2}+a_{hwz}^{2}}\;({\mathrm{ms}}^{-2})$$

The output of the *W_p_* curve were the weighted *a_px_, a_py,_* and *a_pz_* accelerations, and the VTVs (*a_pv_*, ms^−2^) were calculated, as r.m.s (4).

(4) $$a_{pv}=\sqrt{a_{px}^{2}+a_{py}^{2}+a_{pz}^{2}}\;({\mathrm{ms}}^{-2})$$

The vibration daily exposure *A(8)* (ms^−2^) was calculated for both the machine types and then compared with the daily exposure action value (2.5 ms^−2^) and with the daily limit value (5 ms^−2^), according to the European Directive 2002/44/EC (5).

(5) $$A(8)=a_{hv}\sqrt{\frac{T}{8}}\;({\mathrm{ms}}^{-2})$$

where *T* (h) was the daily use of the vibrating machine, and 8 was the daily working hours (according to [1]). The daily vibration exposure value *E_p,d_* was calculated for the vascular risk of the hand-arm system [26] using Equation (6), and it was compared with the daily exposure threshold for the onset and continuing development of vibration white finger [29].

(6) $$E_{p,d}=\sqrt{a_{pv}^{2}T}\;({\mathrm{ms}}^{-1.5})$$

The calculation of *a_v_*, *a_hv,_* and *a_pv_* was carried out using 1/7 of the machines use at LES and 6/7 at HES [30]. The daily exposures were calculated using 3 h of working time per day for blowers and 1 h for mist blowers [30].

### 2.3. Noise

#### 2.3.1. Measurement Chain

Noise levels were measured with a Larson Davis model 831 sound analyzer, equipped with its own microphone and amplifier (Larson Davis, Provo, UT, USA). The instrument recorded both the equivalent continuous sound pressure level and one-third-octave band frequency spectra, in the range 20 Hz–20 kHz with the A-weighting curve. The measurement system was Class 1 compliant [31]. The calibration was performed before and after every measurement cycle using a Brüel & Kjær, model 4230 (Brüel & Kjær, Nærum, Denmark). Variations between references values were smaller than 0.5 dB.

#### 2.3.2. Noise Measurement

The sound pressure levels of the machines at the operator’s ears in the workstation were acquired according to [5]. The duration of each measurement was about one min (the level was constant and repeatable). The microphone was located 0.20 m ± 0.02 m on both sides of the center plane of the operators’ head, with its axis parallel to the operators’ line of vision. Equation (7) was used for the calculation of *L_Aeq_*.

(7) $$L_{Aeq}=10\log\left(\frac{\frac{1}{T}\int_{t1}^{t2}p_{A}^{2}(t)dt}{p_{0}^{2}}\right)\;(\mathrm{dB}(A))$$

where *p_A_* was the weighted sound pressure (Pa), *p*_0_ (20 μPa) was the reference value, *t*_1_ = 0, and *t*_2_ = 60 (s).

Measurements were carried out at LES and at HES: three measurements of sound pressure levels were carried out for each condition. 

#### 2.3.3. Daily Exposure

Equation (8), as defined in [2], was used to calculate the maximum daily exposure time *T* (h) under the condition of *L_Aeq_* higher than the daily exposure limit value (87 dB(A)).

(8) $$T=8\left(10^{\left(\frac{87-L_{Aeq}}{10}\right)}\right)\;(h)$$

where *L_Aeq_* was the acoustic pressure level measured at the operators’ ears (and used in Equation (8) when it was higher than 87 dB(A)).

### 2.4. Statistical Analysis

Data were elaborated by IBM SPSS Statistics (V. 25, International Business Machines Corporation, Armonk, New York, NY, USA). The *t*-test of Student was used to assess significant similarities or differences between the operators and the two groups of machines (mist blowers and blowers) for both the vibration and the noise analysis. The bootstrap utility was used for obtaining robust estimates of standard errors and confidence intervals for the means estimates because the samples were small and could cause heteroscedasticity problem [32]. Even though the bootstrap methodology is computer intensive, it does not require population normality for the mean or other parameters estimation [33]. All the tests were carried out with *p* = 0.01.

## 3. Results

### 3.1. Back Vibration

#### 3.1.1. VTVs Analysis: Unweighted and *W_c_* Weighted Accelerations

The VTVs transmitted to the operators’ back were analyzed using both the unweighted data and the values obtained by the *W_c_* weighting curve [3]. The bootstrap procedure applied to the descriptive statistics of the two machines sets (mist blowers and blowers) gave good results, with very low bias and standard errors, confirming the good stability of the calculated means (Table 3). There was always a difference between the VTVs of mist blowers and blowers, the former higher than the latter, both as unweighted data and after the application of the *W_c_* weighting curve (Table 3). This difference was confirmed by the comparison of the mean values using the *t*-test of Student (sign < 0.01). Any difference was revealed by the *t*-test of Student in the comparison of the mean VTVs among the operators (sign > 0.01).

**Figure 4:**
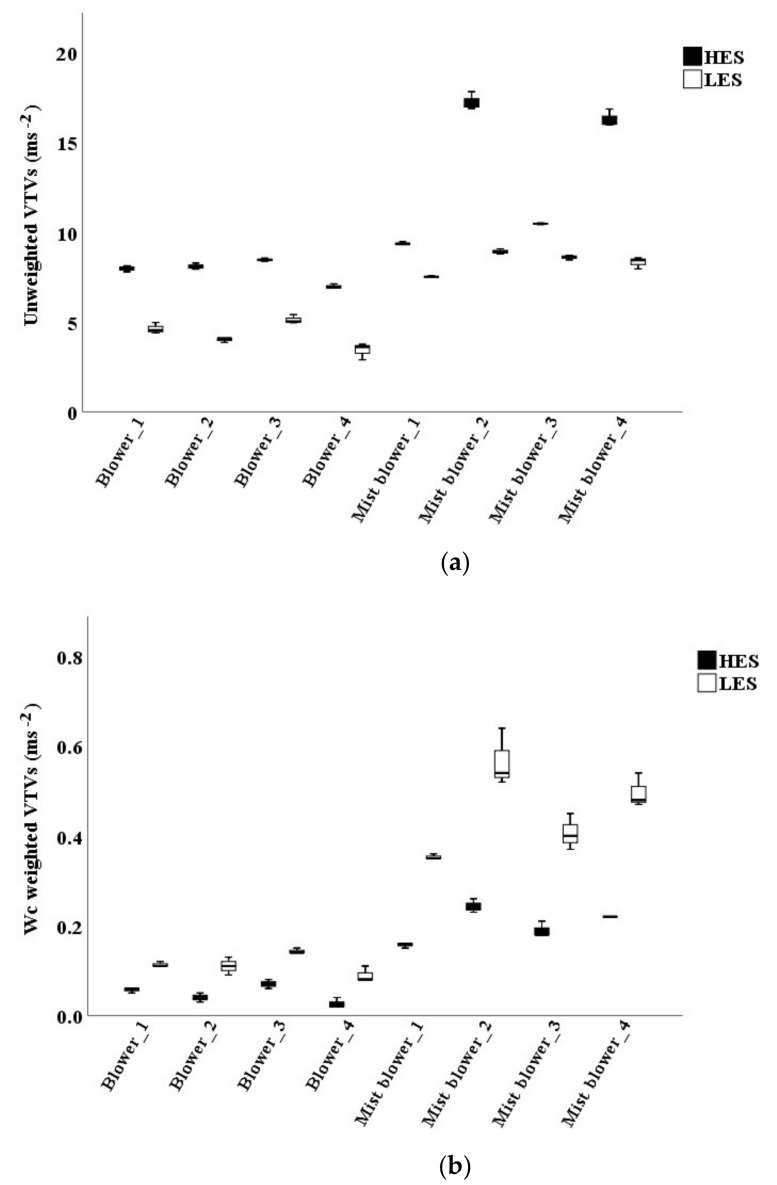
Vibration total values (VTVs) on the operators’ back of mist blowers and blowers: unweighted (**a**) and *W_c_* weighted (**b**). LES: low engine speed; HES: high engine speed.

Mist blowers #2 and #4 showed the highest values with unweighted VTVs at HES (Figure 4a). When using the *W_c_* weighting curve, the VTVs of the mist blowers ranged from 0.4 to 0.67 ms^−2^ at LES, and they were around 0.22 ms^−2^ at HES. The VTVs of the blowers never exceeded 0.17 ms^−2^ (Figure 4b). The highest VTV *W_h_* weighted (considering 1/7 of the machines use at LES and 6/7 at HES) was reached by mist blower #2 (0.38 ms^−2^), the lowest by blower #2 (0.04 ms^−2^).

#### 3.1.2. Back Unweighted Acceleration Analysis: *x*, *y*, and *z*-Axes

**Figure 5:**
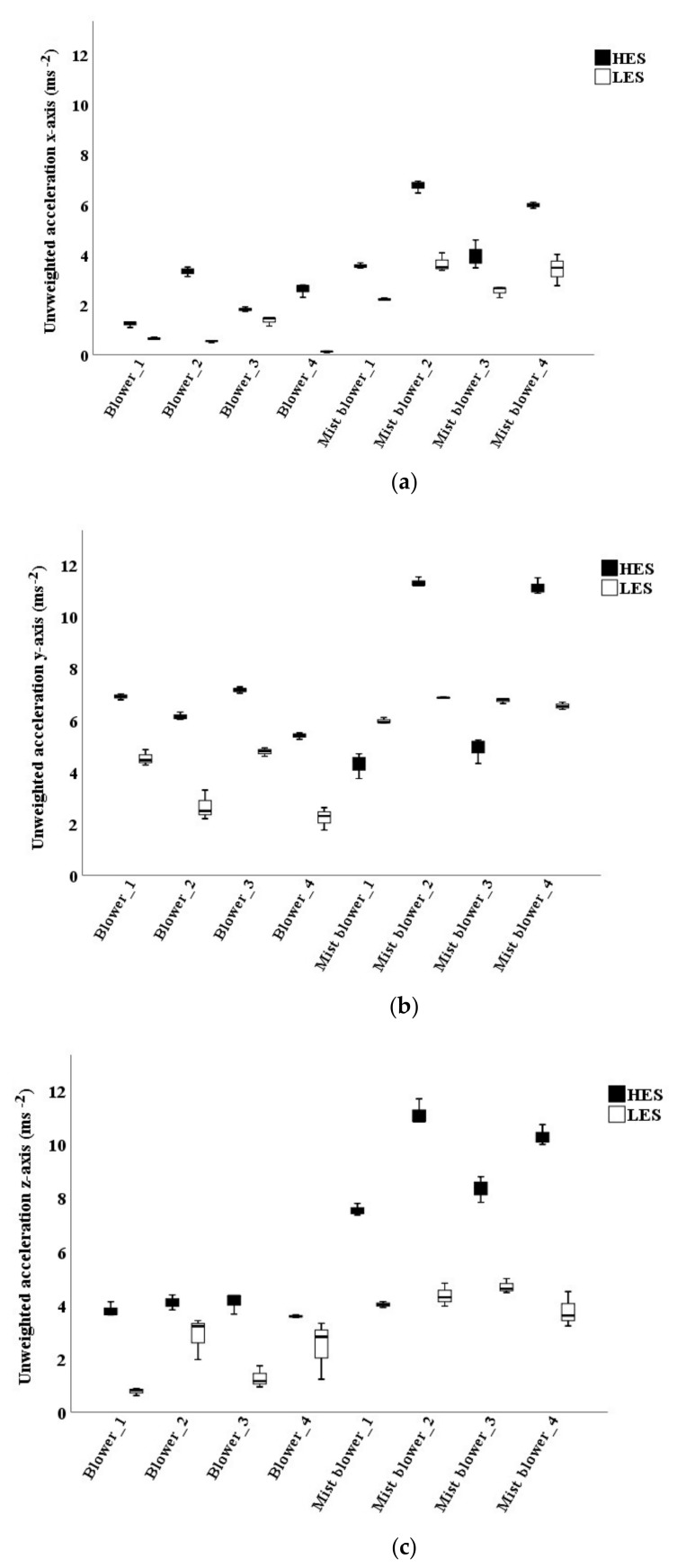
Unweighted accelerations measured on the operators’ back along the axes. (**a**) *x*-axis; (**b**) *y*-axis; (**c**) *z*-axis. LES: low engine speed; HES: high engine speed.

ISO 2631-1 uses only the *W_c_* curve in the health evaluation and does not concern *y* and *z*-axes: for this reason, only the unweighted acceleration along the three axes was studied. The highest mean values were observed along *y* and *z*-axes for both the machine types at LES and at HES (mist blowers #2 and #4 had values higher than 11 ms^−2^). The mean x values were the lowest (Figure 5).

#### 3.1.3. Frequency Analysis

**Figure 6:**
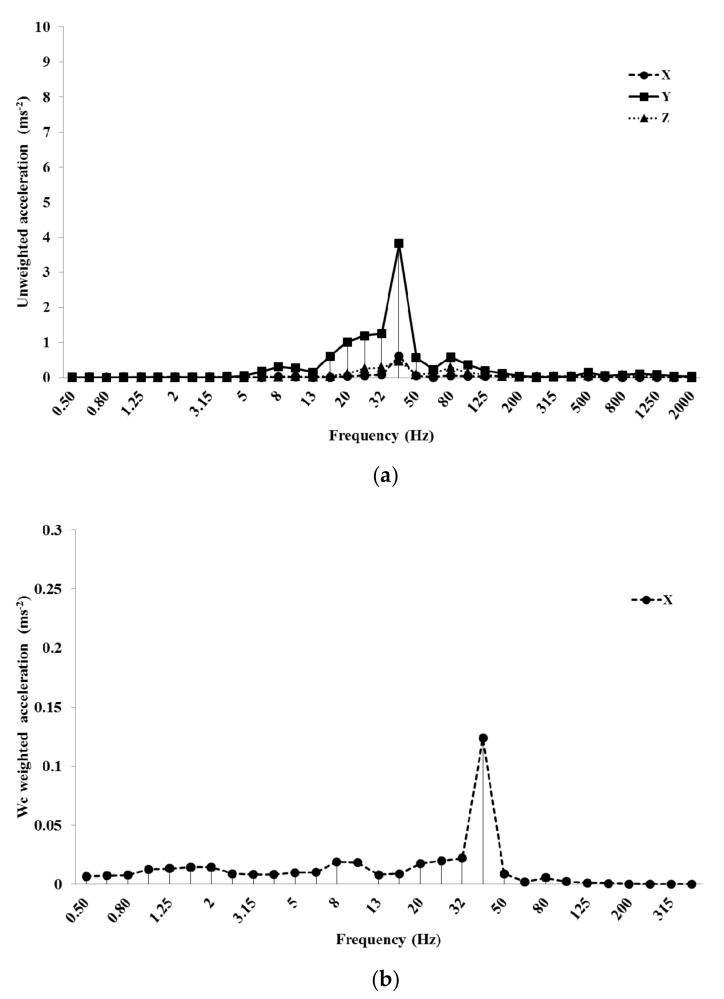
Unweighted (**a**) and *W_c_* weighted frequency analysis (**b**) of the acceleration values of blower #1 at LES (low engine speed).

All the examined machines had the fundamental harmonic between 40 and 50 Hz at LES and between 100 and 116 Hz at HES. This harmonic was produced by the engine rotation speed (between 2200 and 3000 rpm at LES and between 6200 and 7000 rpm at HES). Blower #1 at LES (at 40 Hz) had the highest unweighted value along the *y*-axis (3.82 ms^−2^), the lowest along the *x*-axis (0.62 ms^−2^) (Figure 6a). This value was cut to 0.12 ms^−2^ after the use of the *W_c_* weighting curve (Figure 6b).

**Figure 7:**
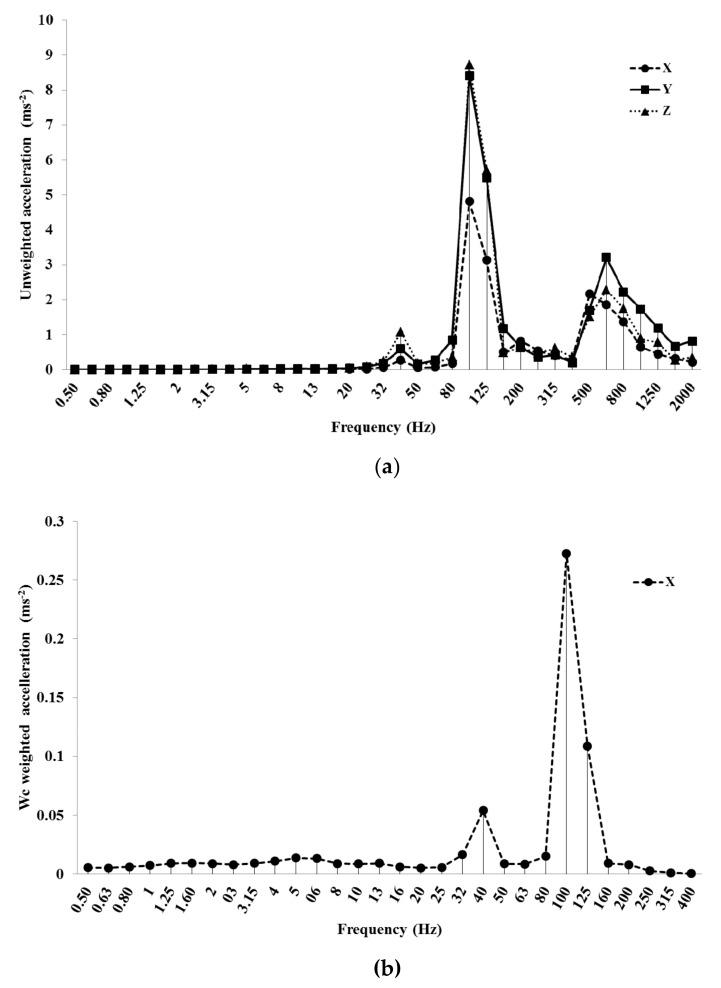
Unweighted (**a**) and *W_c_* weighted frequency analysis (**b**) of the acceleration values of the mist blower #2 at HES (high engine speed).

Mist blower #2 at HES showed 8.5 ms^−2^ along *y* and *z*-axes and 4.5 ms^−2^ along the *x*-axis in the unweighted acceleration, at the fundamental harmonic (100 Hz, Figure 7a). With the *W_c_* weighting curve, the *x*-axis value dropped down to 0.27 ms^−2^ (Figure 7b). 

Figure 6b and Figure 7b do not show data after 400 Hz (the *W_c_* weighting curve is applied only along the *x*-axis until this frequency).

### 3.2. HAV

#### 3.2.1. VTVs (Unweighted, *W_h_* and *W_p_* Weighted)

Mist blowers and blowers showed the highest mean VTVs with both the unweighted and the *W_p_* weighting curves at HES.

It is worthy of note the high values when using the *W_p_* weighting curve: 7.70 (LES) and 13.81 (HES) ms^−2^ in mist blowers, 6.64 (LES) and 12.09 (HES) ms^−2^ in blowers, very close to the unweighted data (Table 4). The *W_h_* weighting curve showed the highest mean VTVs at LES: 3.26 ms^−2^ (mist blowers) and 2.53 ms^−2^ (blowers, Table 4).

The bias obtained by the bootstrap test was acceptable: the highest values were observed in the blowers at HES (0.02 and 0.03), respectively, in the unweighted data and with the use of the *W_p_* weighting curve. This is probably due to some fluctuations in the acceleration values among different models of blowers. The *t*-test of Student did not detect differences among the average VTVs in the operators (sign > 0.01), while revealed a difference among the average VTV in the mist blowers and blowers only at LES (sign < 0.00).

**Figure 8:**
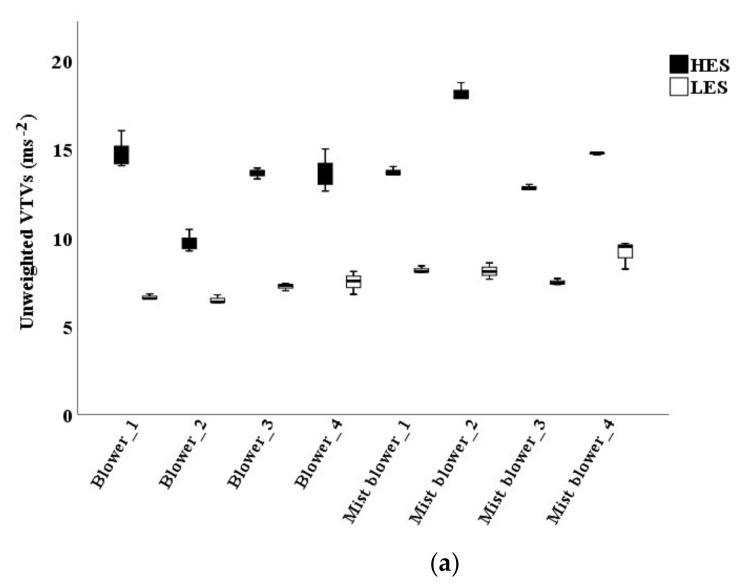

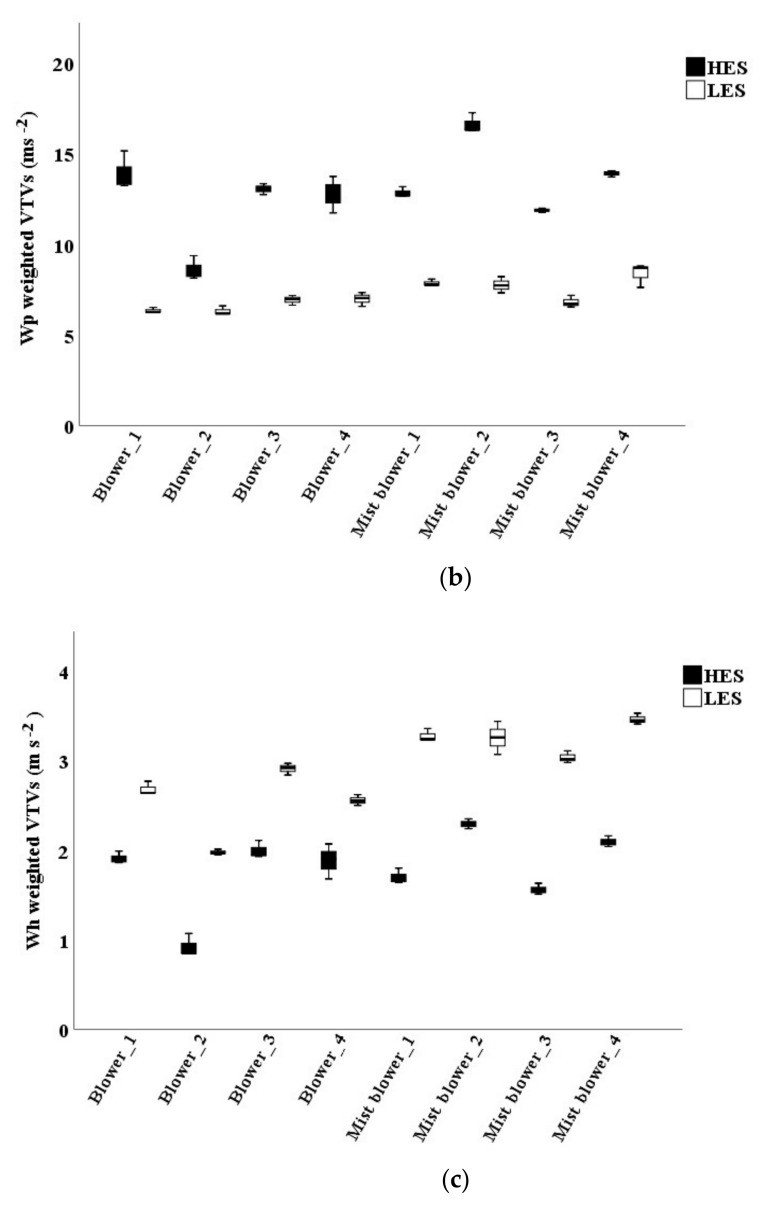
Vibration total values (VTVs) of the hand-arm system of the signals unweighted (**a**), *W_p_* weighted (**b**), and *W_h_* weighted (**c**).

The highest acceleration values were observed at HES with unweighted data (Figure 8a) and with the use of the *W_p_* curve (Figure 8b): the two graphs are very similar. 

For this reason, from now on, the unweighted data are not discussed. The use of the *W_h_* curve showed the highest values at LES: 3 ms^−2^ and more (until 2.7 ms^−2^ at HES, Figure 8c). The highest VTVs *W_h_* weighted (considering 1/7 of the machines use at LES and 6/7 at HES) were 2.51 ms^−2^ (mist blower #2) and 2.10 ms^−2^ (blower #1). The highest daily exposure value *A(8)* was 0.89 ms^−2^ in mist blowers and 1.29 ms^−2^ in blowers. The highest *E_p,d_* was 958 ms^−1.5^ in the mist blower #2 and 1446 ms^−1.5^ in the blower #1. 

#### 3.2.2. Accelerations Along *x*, *y*, and *z*-axes

VTVs were low. The *W_h_* weighting curve showed the highest values along the *z*-axis at LES for mist blowers (2.45 ms^−2^), quite close to the data of the *y*-axis. At HES, the highest values were along the *x* (mist blowers) and *y* (blowers) axes. The *W_p_* weighting curve showed the lowest accelerations along the *z*-axis in both the machines. The highest values were observed along the *x* (blowers) and *y* (mist blowers) axes (around 10 ms^−2^).

#### 3.2.3. Frequency Analysis

**Figure 9:**
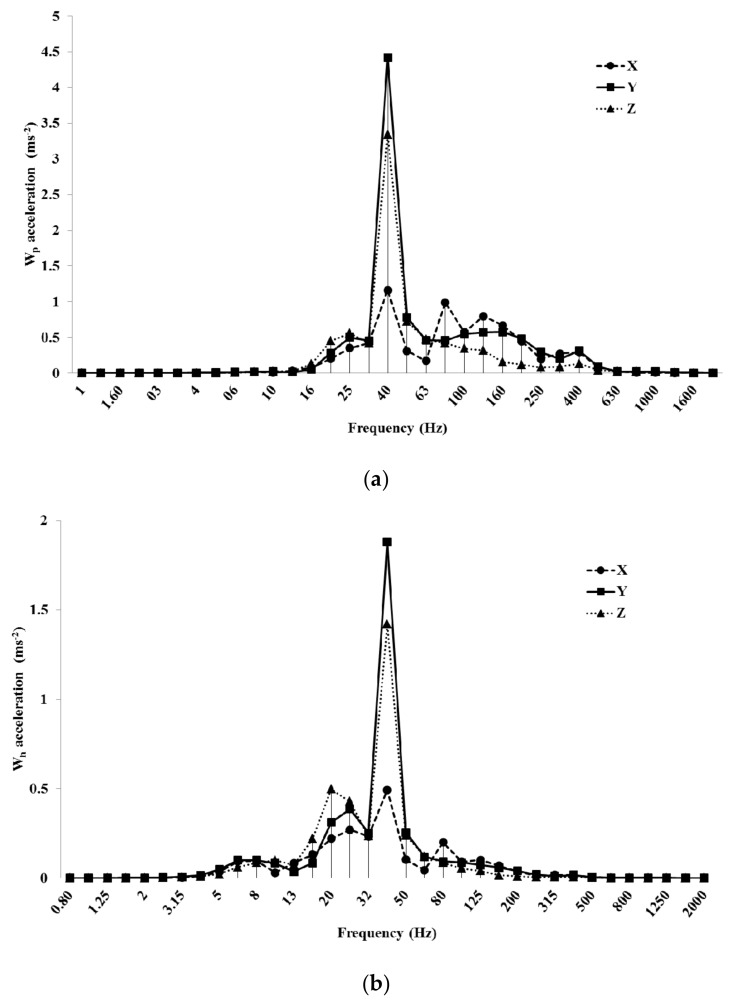
Frequency analysis of blower #1 acceleration values at LES (low engine speed): *W_p_* (**a**) and *W_h_* (**b**) weighting curves.

The frequency analysis regarding the same machines analyzed the back acceleration: blower #1 at LES and mist blower #2 at HES. The former showed the fundamental harmonic at 40 Hz: here, with the *W_p_* weighting curve, the value along the *y*-axis was about 4.5 ms^−2^ (Figure 9a), lowering to 1.8 ms^−2^ with the *W_h_* weighting curve (Figure 9b). 

**Figure 10:**
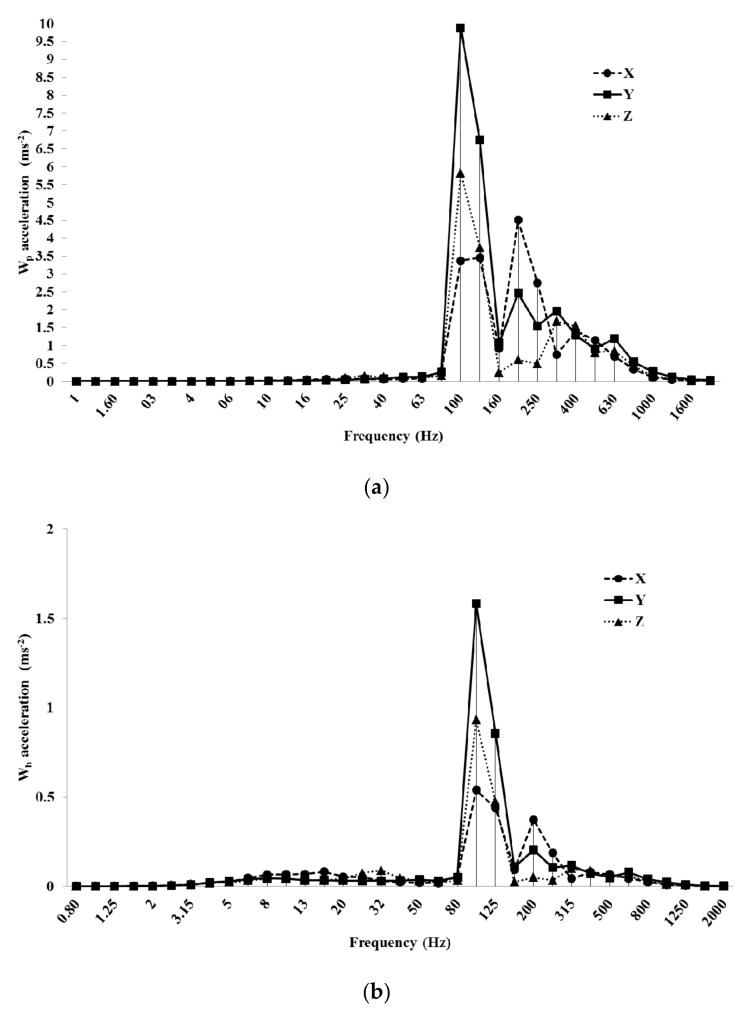
Frequency analysis of acceleration values with *W_p_* (**a**) and *W_h_* (**b**) weighting curves in the mist blower #2 at HES (high engine speed) condition.

The frequency analysis of mist blower #2 at HES showed the fundamental harmonic always at 100 Hz (Figure 10). The highest value was along the *y*-axis (almost 10 ms^−2^ in the *W_p_* curve and 1.6 ms^−2^ in the *W_c_* curve, Figure 10). The second harmonic was at 200 Hz, with the highest value along the *x*-axis (4.7 ms^−2^ in the *W_p_* curve and 0.4 ms^−2^ in the *W_c_*). 

### 3.3. Noise

The noise analysis logarithmically studied the acoustic pressure levels *L_Aeq_*.

The values at HES (exceeding 100 dB(A) in mist blowers) were higher than at LES, where data never overcame 83.7 dB(A).

As expected, there were no differences among the operators (the measured pressure levels were constant), statistically confirmed by the *t*-test of Student (sign > 0.01). The only little difference was between the left and the right ears, the second being slightly higher because the operators were right-handed (Table 5).

Mist blowers had the highest values, both at LES and at HES. The standard deviation was low in mist blowers, and high in blowers (reaching 8.34 dB(A) at the right ear at the HES), confirming the presence of some variations in the sound pressure values acquired at the operators’ ear by different blower types (Table 5).

**Figure 11:**
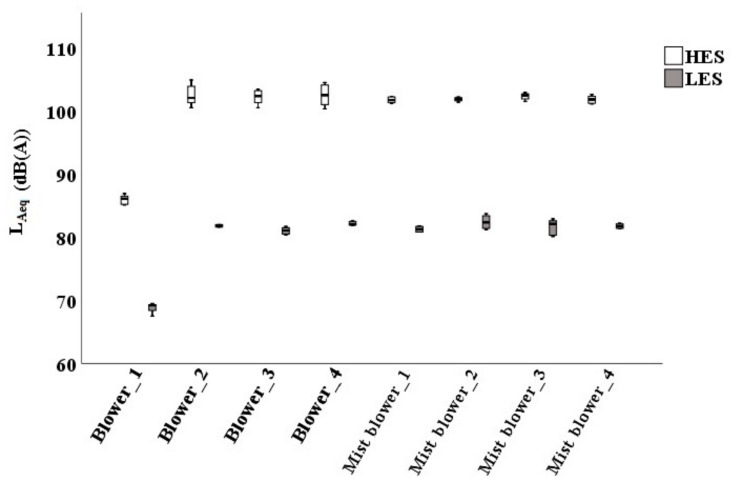
Noise pressure levels at the operators’ ear in all the examined machines at LES (low engine speed) and at HES (high engine speed).

Figure 11 shows the lowest *L_Aeq_* values in the blower #1, both at LES (68.7 dB(A)) and at HES (85.8 dB(A)). Blower #3 had instead a mean of 81.1 dB(A) at LES and 102.1 dB(A) at HES. 

#### Noise Frequency Analysis

**Figure 12:**
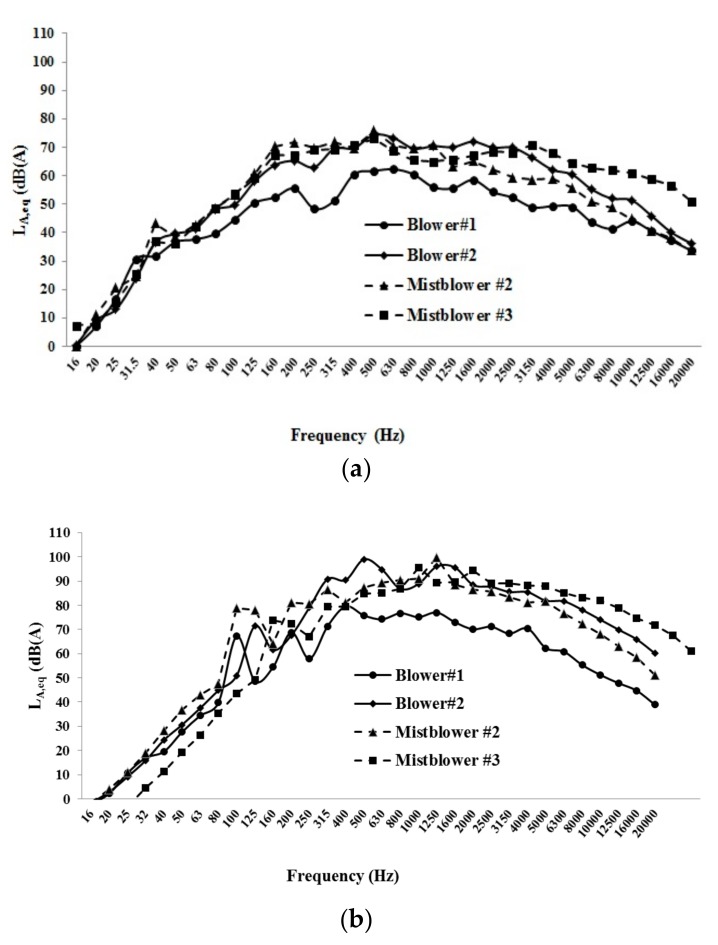
Sound pressure levels of blower #1, blower #2, mist blower #2, and mist blower #3 at LES (low engine speed) (**a**) and at HES (high engine speed) (**b**).

The frequency analysis considered, as an example, the sound pressure levels of blower #1, blower #2, mist blower #2, and mist blower #3, both at LES (Figure 12a) and at HES (Figure 12b).

With the exception of the blower #1, all the machines showed homogeneous shapes, with the highest values at HES (in the interval 85–100 dB(A)) between 500 and 1600 Hz.

## 4. Discussion

Mist blowers showed VTVs higher than blowers also after the application of the *W_c_* weighting curve in the analysis of the back vibration. This is probably due both to the different revolutions per minute of the engines (see Table 2) and to the presence of the tank with the pesticide in the mist blowers, that could unbalance the machine on the operators’ back, as observed also by Calvo et al. [24].

The mean VTVs did not, however, appear dangerous for the vibration transmitted to the operators’ back, according to the ISO 2631-1 health condition, which accounts only the fore-and-aft direction (*x*-axis). The highest observed values were in the mist blower #2 (0.64 ms^−2^ at LES and 0.26 ms^−2^ at HES) and in the blower #3 (0.15 ms^−2^ at LES and 0.08 ms^−2^ at HES). The daily exposures values *A(8)* were 0.31 ms^−2^ and 0.09 ms^−2^, respectively, for mist blowers and blowers. These values were both lower than the daily exposure action value (0.5 ms^−2^) and, therefore, they are not legally dangerous for the operators’ health. In this case, however, the ISO 2631-1 standard is not exhaustive, because it does not consider y and *z*-axes (respectively, the shoulder-shoulder and the buttocks-head directions). In this work, the unweighted values along these two axes were indeed quite high at HES (between 10 and 12 ms^−2^ in mist blowers #2 and #4), but they reached only 6 ms^−2^ along the *x*-axis.

The use of the *W_h_* weighting curve for the HAV evaluation (as requested by the ISO 5349-1) produced the highest *A(8)* values in the mist blower #2 (2.52 ms^−2^) and in the blower #1 (2.10 ms^−2^), but only the former is slightly higher than the daily exposure action value (2.5 ms^−2^, [1]). The European Directive 2002/44 [1] permits the use of these machines without special attention, but recent studies ([29,34]) highlighted that for specific exposures (frequencies between 20 and 200 Hz), the use of the weighting curve proposed by the ISO 5349-1 might miss the vascular risk, cause of the Reynaud phenomenon. The ISO/TR 18570 standard should solve this problem, thanks to a new weighting curve (*W_p_*) and to a new parameter (*E_p,d_*), used as complimentary of (and not instead of) the ISO 5349-1. The *W_p_* weighting curve and the *E_p,d_* parameters were introduced on the basis of experimental and epidemiological studies [35]. In this work, the highest *E_p,d_* obtained by the *W_p_* weighting curve were 958 ms^−1.5^ in mist blower #2 and 1446 ms^−1.5^ in blower #1, the second considered critical for the risk of vibration-induced white finger [29,36].

The highest value in blower #1 was not due to high vibration values (13.92 ms^−2^ versus 15.97 ms^−2^ of the mist blower #2) but to the daily use (3 h versus 1 h for mist blowers, according to the CEN/TR 15350 [30]). In this case, the *E_p,d_* parameter was heavily conditioned by the duration of the daily exposure, as observed also in other works [14,37,38]. The reduction of the duration of exposure is sometimes more effective than reducing the vibration magnitude [39].

**Figure 13:**
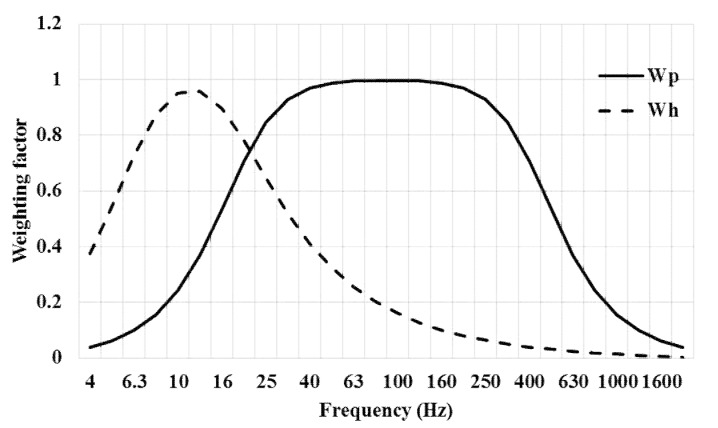
The weighting curves *W_h_* and *W_p_*.

Another comment concerns the *W_h_* weighting curve. The *W_h_* weighting curve decreases the acceleration data in the range 40–400 Hz and resets all the acceleration after 400 Hz (Figure 13). The *W_p_* weighting curve, instead, does not reduce the unweighted acceleration values between 40 and 250 Hz (Figure 13), the interval in which the highest accelerations were observed in this work. These machines are, therefore, not dangerous for bones, joints, and muscles, but some risk may arise for the vascular system.

The noise was another negative component, not only by itself but also because it may condition the perception of the received vibration, as studied by Huang and Griffin [40].

Blower #1 had the lowest *L_Aeq_* values both at LES (68.7 dB(A)) and at HES (85.8 dB(A)). The reason was in some special features of blower #1: the exhaust was inside the blower hose, and a special plastic covered the engine and the fan. This good solution for the lowering of the emitted noise might, however, cause environmental problems, as the increase of CO, HC, NO_x_, SO_x_, and PM_x_ in the air flowed at high speed through the blower hose which may aggravate the pollution in the surrounding areas. 

The average sound pressure levels of the other blowers were 81.7 dB(A) at LES and 102.6 dB(A) at HES, slightly higher than that reported by Calcante et al. [7] (72.6 dB(A) at LES and 97.3 at HES) and Silva et al. [19] (81.6 dB(A)), and in line with Calvo et al. [14] (96.4 dB(A)) and Pasanen et al. [20] (from 92 to 102 dB(A)).

Mist blowers showed mean values of 81.8 dB(A) at LES and 101.92 at HES, close to the values obtained by Sasaki et al. [21] and Vilela et al. [23] (about 100 dB(A) at HES).

These machines are used for almost all the time at HES (about 6/7 of the total time, as in CEN/TR 15350), and in this work, all of them exceeded 85 dB(A): for this reason, ear protectors are mandatory, as well as periodic medical check-up and training and workers’ information, to avoid permanent ear damages. Using the acoustic pressure levels measured in this work, without ear protectors an operator could not use a mist blower for more than 15 min per day and a blower for more than 14 min per day (with the exclusion of the blower #1) to not overcome the daily exposure limit (87 dB(A)). Operators are protected if they wear ear protectors, but the matter of the high noise levels for the nearby people remains.

## 5. Conclusions

Back and hand-arm vibration produced by the examined blowers and mist blowers did not seem dangerous for the operators’ health. Mist blowers showed the highest VTVs at the back (0.38 ms^−2^) and, also considering an unlikely daily use of 8 h, the VTVs remain lower than the daily action value (0.5 ms^−2^), according to the European Directive 2002/44. The criticisms observed in this work were the high unweighted values along *y* and *z*-axes, not considered by the ISO 2631-1 health procedure for the back vibration. The evaluation along these axes should be accounted for in a future revision of this standard.

Using the ISO 5349-1 for the hand-arm system, the HAV values were always lower than the daily exposure action value (2.5 ms^−2^), with only one little exception (2.51 ms^−2^ in the mist blower #2). These machines do not appear dangerous for the operators’ hand-arm system, but with the use of the *W_p_* weighting curve (as proposed by the ISO/TR 18570 standard), the values increased, especially in the blower #1, that showed values higher than the daily exposure threshold for the onset of the vibration white finger. These values were not due to a high VTV, but to the daily use of blowers (3 h versus 1 h of mist blowers), increasing the risk of the criticality of the parameter ‘duration of daily exposure’ in the HAV white finger analysis.

Noise pressure levels were higher than 100 dB(A) at HES in almost all the machines, with the exception of the blower #1 that had the exhaust inside the blower pipe. If the blowers #2, #3, and #4 could not be used without ear protectors for more than 15–20 min per day, the blower #1 could increase the dust and the exhaust in the surrounding. Blowers are spread in the maintenance of public gardens and urban parks, and gas emitted through the blower hose at high speed may increase the number of harmful emissions, more dangerous especially in the cold season, when these machines are most commonly used for the removal of the dead leaves.

## Figures and Tables

**Table 1:** Operators’ characteristics.

Operator	Age	Mass	Height	Operator	Age	Mass	Height
		kg	m			kg	m
#1	38	81	1.72	#6	48	83	1.75
#2	43	86	1.85	#7	52	84	1.78
#3	29	75	1.73	#8	26	79	1.84
#4	41	83	1.81	#9	44	84	1.79
#5	28	78	1.80	#10	50	82	1.69

**Table 2:** Technical characteristics of the machines.

Machine	Production	Displacement	Mass	Rotation Speed (LES)	Rotation Speed (HES)	Power	Tank Capacity	Air Volume at Nozzle **
	Year	cm^3^	kg	rpm *	rpm *	kW	cm^3^	m^3^ h^−1^
Mist blower #1	2010	77	12.2	2400	6160	3.6	11,000	-
Mist blower #2	2006	77	12.2	2400	6200	3.6	17,000	-
Mist blower #3	2015	72.4	12.5	2700	6100	3.7	14,000	-
Mist blower #4	2016	63.3	12.8	3000	6800	2.9	13,000	-
Blower #1	2010	77	12.2	2400	6160	3.6	-	1100
Blower #2	2006	77	12.2	2400	6600	3.6	-	1140
Blower #3	2016	63.3	10.8	2500	6900	2.9	-	1300
Blower #4	2015	65.6	11.2	2200	7000	2.9	-	1320

* rpm: revolutions per minute; ** Air volume at nozzle: nominal values. LES: low engine speed; HES: high engine speed.

**Table 3:** The bootstrap method (1000 bootstrap samples) applied to the vibration total values (VTVs) (unweighted and after the application of *W_c_* weighting curve) produced by mist blowers and blowers on the operators’ back.

	Machine		LES (ms^−2^)					HES (ms^−2^)				
			Statistic	Bias	SE	Lower	Upper	Statistic	Bias	SE	Lower	Upper
Unweighted	M	Mean	8.33	−0.01	0.15	8.06	8.58	13.34	0.03	1.02	11.24	15.71
		SD	0.56		0.08	0.42	0.64	3.63		0.3	3.3	3.66
	B	Mean	4.29	0.00	0.19	3.88	4.68	7.87	0.00	0.17	7.47	8.21
		SD	0.72		0.12	0.51	0.83	0.58		0.11	0.4	0.67
*W_c_* curve	M	Mean	0.45	0.00	0.02	0.41	0.5	0.2	0.00	0.01	0.18	0.22
		SD	0.09		0.01	0.06	0.1	0.03		0.00	0.02	0.04
	B	Mean	0.11	0.00	0.00	0.1	0.12	0.04	0.00	0.00	0.03	0.05
		SD	0.02		0.00	0.01	0.02	0.01		0.00	0.01	0.02

M: mist blowers; B: blowers; SD: standard deviation; SE: standard error; Lower and Upper: 95% confidence interval. LES: low engine speed; HES: high engine speed.

**Table 4:** The bootstrap method (1000 bootstrap samples) applied to the hand-arm vibration total values (VTVs) (unweighted and with the application of the *W_h_* and *W_p_* weighting curves).

	Machine		LES (ms^−2^)					HES (ms^−2^)				
			Statistic	Bias	SE	Lower	Upper	Statistic	Bias	SE	Lower	Upper
Unweighted	M	Mean	8.21	−0.01	0.21	7.85	8.61	14.86	0.00	0.59	13.87	15.94
		SD	0.74		0.16	0.43	0.86	1.13		0.40	1.49	2.44
	B	Mean	6.95	0.00	0.15	6.68	7.24	12.95	0.02	0.60	11.57	14.08
		SD	0.53		0.11	0.34	0.64	2.15		0.42	1.47	2.46
*W_h_* curve	M	Mean	3.26	0.00	0.05	3.16	3.36	1.91	0.00	0.09	1.75	2.06
		SD	0.18		0.02	0.15	0.20	0.31		0.04	0.27	0.33
	B	Mean	2.53	0.00	0.10	2.31	2.73	1.68	0.01	0.13	1.38	1.94
		SD	0.36		0.06	0.25	0.42	0.47		0.10	0.26	0.55
*W_p_* curve	M	Mean	7.70	0.00	0.20	7.32	8.05	13.81	0.00	0.52	12.90	14.76
		SD	0.70		0.12	0.52	0.78	1.87		0.33	1.34	2.14
	B	Mean	6.64	0.00	0.11	6.43	6.85	12.09	0.03	0.63	10.64	13.27
		SD	0.40		0.06	0.30	0.45	2.26		0.47	1.50	2.60

M: mist blowers; B: blowers; SD: standard deviation; SE: standard error; Lower and Upper: 95% confidence interval. LES: low engine speed; HES: high engine speed.

**Table 5:** The bootstrap method (1000 bootstrap samples) applied to the *L_Aeq_* data at the operators’ ears (left and right) using mist blowers and blowers at LES (low engine speed) and at HES (high engine speed).

Engine Speed	Ear	Machine	Statistic	Bias	SE		Lower	Upper
			*L_Aeq_* (dB(A))					
LES	L	M	Mean	81.10	0.00	0.15	80.75	81.37
			SD	0.52	−0.04	0.10	0.35	0.60
		B	Mean	78.30	−0.01	1.76	74.73	81.68
			SD	6.04	−0.43	1.40	3.36	7.03
	R	M	Mean	82.50	0.00	0.20	82.11	82.87
			SD	0.69	−0.04	0.11	0.51	0.80
		B	Mean	78.60	−0.01	1.63	75.11	81.71
			SD	5.61	−0.43	1.39	3.26	6.46
HES	L	M	Mean	101.60	0.00	0.13	101.39	101.90
			SD	0.45	−0.02	0.06	0.37	0.48
		B	Mean	97.40	−0.02	1.89	93.44	101.03
			SD	6.53	−0.48	1.58	3.79	7.57
	R	M	Mean	102.20	0.00	0.14	101.92	102.46
			SD	0.47	−0.03	0.08	0.35	0.53
		B	Mean	99.10	−0.02	2.42	94.02	103.74
			SD	8.34	−0.61	2.00	4.92	9.66

M: mist blowers; B: blowers; L: left ear; R: right ear, SD: standard deviation; SE: standard error; Lower and Upper: 95% confidence interval.
